# Dysregulation of Transcription Factor Activity During Formation of Cancer-Associated Fibroblasts

**DOI:** 10.3390/ijms21228749

**Published:** 2020-11-19

**Authors:** Przemysław Kapusta, Joanna Dulińska-Litewka, Justyna Totoń-Żurańska, Agnieszka Borys, Paweł S. Konieczny, Paweł P. Wołkow, Michał T. Seweryn

**Affiliations:** 1 Center for Medical Genomics OMICRON, Jagiellonian University Medical College, 31-034 Kraków, Poland; przemyslaw.kapusta@uj.edu.pl (P.K.); justyna.toton-zuranska@uj.edu.pl (J.T.-Ż.); agnieszka.borys@uj.edu.pl (A.B.); pawel.konieczny@outlook.pl (P.S.K.); 2 Chair of Medical Biochemistry, Jagiellonian University Medical College, 31-034 Kraków, Poland; joanna.dulinska-litewka@uj.edu.pl; 3 Department of Pharmacology, Jagiellonian University Medical College, 31-531 Kraków, Poland; 4 Faculty of Mathematics and Computer Science, University of Łódź, 90-238 Łódź, Poland

**Keywords:** cancer-associated fibroblasts (CAFs), prostate cancer, long non-coding RNA (lncRNA), *FER1L4*, transcription factors (TFs)

## Abstract

The reciprocal interactions between cancer cells and the quiescent fibroblasts leading to the activation of cancer-associated fibroblasts (CAFs) serve an important role in cancer progression. Here, we investigated the activation of transcription factors (TFs) in prostate fibroblasts (WPMY cell line) co-cultured with normal prostate or tumorous cells (RWPE1 and RWPE2 cell lines, respectively). After indirect co-cultures, we performed mRNA-seq and predicted TF activity using mRNA expression profiles with the Systems EPigenomics Inference of Regulatory Activity (SEPIRA) package and the GTEx and mRNA-seq data of 483 cultured fibroblasts. The initial differential expression analysis between time points and experimental conditions showed that co-culture with normal epithelial cells mainly promotes an inflammatory response in fibroblasts, whereas with the cancerous epithelial, it stimulates transformation by changing the expression of the genes associated with microfilaments. TF activity analysis revealed only one positively regulated TF in the RWPE1 co-culture alone, while we observed dysregulation of 45 TFs (7 decreased activity and 38 increased activity) uniquely in co-culture with RWPE2. Pathway analysis showed that these 45 dysregulated TFs in fibroblasts co-cultured with RWPE2 cells may be associated with the RUNX1 and PTEN pathways. Moreover, we showed that observed dysregulation could be associated with *FER1L4* expression. We conclude that phenotypic changes in fibroblast responses to co-culturing with cancer epithelium result from orchestrated dysregulation of signaling pathways that favor their transformation and motility rather than proinflammatory status. This dysregulation can be observed both at the TF and transcriptome levels.

## 1. Introduction

Interactions between cancer cells and cancer-associated fibroblasts (CAFs) play an important role in promoting tumorigenesis, extracellular matrix (ECM) remodeling and tumor invasion, as well as the induction of metastasis [1,2]. The molecular mechanisms leading the transition from quiescent fibroblasts to activated CAFs are, however, still unclear. CAFs are characterized by a contractile phenotype, associated with the expression of α-smooth muscle actin (αSMA), similar to activated fibroblasts responsible for wound healing (i.e., myofibroblasts). [3,4]. The well-established activating signals for myofibroblast differentiation are transforming growth factor beta (TGF-β) family ligands, which, in cancer, might promote CAF activation [5,6] and tumor growth and progression [7,8].

Increasing evidence suggests that CAFs are a heterogeneous population and have diverse functions depending on tumor developmental stage and tissue microenvironment [9]. Elyada et al., in their single-cell RNA sequencing experiment, showed the existence of myofibroblastic and inflammatory CAFs in human pancreatic ductal adenocarcinoma tumors, each having unique gene signatures in vivo [10]. Costa et al. used multicolor flow cytometry to identify four subtypes of CAFs associated with breast cancer, with distinct properties and levels of activation [11]. Recent discoveries identified transforming growth factor β (TGF-β) and interleukin 1 (IL-1) as tumor-secreted ligands that promote CAFs heterogeneity [12]. Moreover, it was shown that activation of fibroblasts by cancer cells induced self-sustaining stromal cell-derived factor 1 (SDF1/CXCL12) and TGF-β autocrine signaling pathways to promote the formation of CAFs with increased α-SMA expression levels [13]. The activated TGF-β receptor complex initiates several downstream cascades, including the canonical Smad2/3 signaling pathway and non-canonical pathways, such as phosphoinositide 3-kinases (PI3K/AKT), Mitogen-activated protein kinase (MAPK) pathways (extracellular signal regulated kinase (ERK), Jun kinase (JNK), and p38 MAPK), as well as nuclear factor kappa-light-chain-enhancer of activated B cells (NF-κB) [14,15]. Moreover, transversal signaling at the SMAD level integrates signals from other pathways, e.g., Notch, Wnt/β-catenin, TNF-α and epidermal growth factor receptor (EGFR) [16]. The heterogeneity of the pathways engaged in the conversion of normal fibroblasts into CAFs may be associated with the unique functions of CAF subpopulations in different types of tumors. [17]. This suggests that several cytokines or chemokines may be involved in the conversion of normal fibroblasts into CAFs, and some of these form a feedback loop between cancer cells and CAFs.

Therefore, we sought to perform indirect co-cultures of normal prostate fibroblasts (WPMY1) with either normal (RWPE1) or cancerous (RWPE2) prostate epithelial cells at two time points, to catch a glimpse of the feedback loop between CAFs and normal or tumorous cells. We also set to derive a dynamic landscape of the regulatory activity of transcription factors (TFs) based on the mRNA sequencing at those time points. The results of our work point towards the identification of early and potentially causal pathways in CAF transformation in this unique model.

## 2. Results

### 2.1. mRNA-seq Gene Expression Analysis

The pairwise comparison of WR1 (WPMY1 + RWPE1) and WR2 (WPMY1 + RWPE2) after 6 h co-culture did not show any statistical significance. After 24 h, we found 107 differently expressed (DE) genes—46 downregulated and 61 upregulated in WR1 condition (Supplementary Table S1). In the comparison between the control (CTRL) (WPMY1 alone) and WR2 conditions, after 24 h, we found only one DE gene, *KLF9* (logFC = 1.15, *p* = 1.64 × 10^−8^, adj.*p* = 2.6 × 10^−4^). At the same time, *KLF9* was also overexpressed in the WR1 condition (as compared to the CTRL group) (logFC = 1.19, *p* = 9.57 × 10^−9^, adj.*p* = 7.61 × 10^−5^, Supplementary Material 1). After 24 h, we detected two genes differentially expressed between the WR1 and WR2 groups with false discovery rate (FDR) < 0.1. The first, *MMP3* (logFC = −1.53, *p* = 5.10 × 10^−6^, adj.*p* = 8.05 × 10^−2^), was overexpressed in the WR1 group, whereas the second, *FER1L4* (logFC = 1.34, *p* = 1.01 × 10^−5^, adj.*p* = 8.05 × 10^−2^), was overexpressed in the WR2 group.

**Figure 1:**
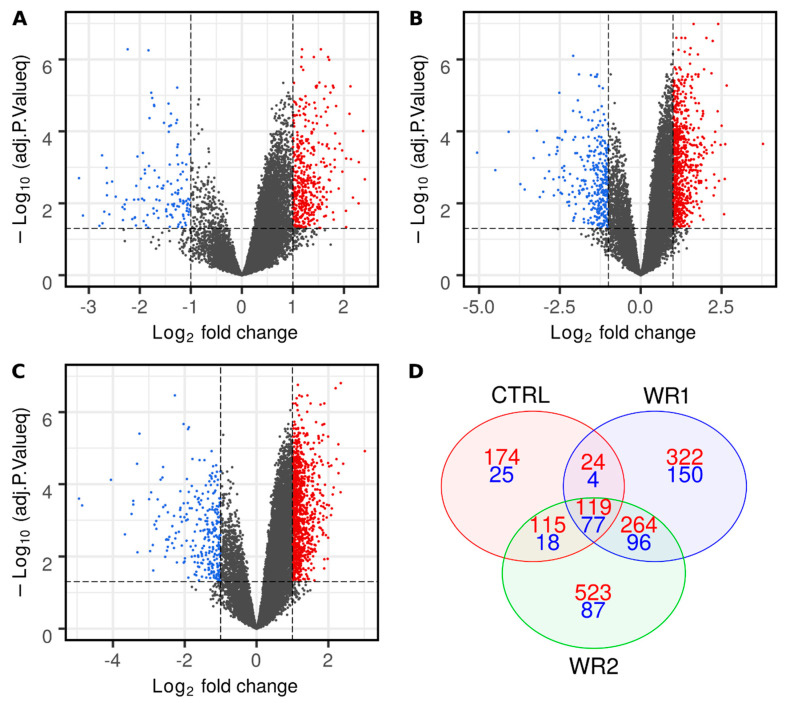
Differentially expressed (DE) gene analysis between 6 and 24 h in each condition separately. Volcano plots represents the distribution of DE genes between 6 and 24 h for the control (CTRL) (**A**), WR1 (**B**) and WR2 (**C**) groups. Lines separate logFC > 1 and adj. *p* value < 0.05. Venn diagram of the distribution of DE genes between 6 and 24 h in each condition with logFC > 1 (**D**). Red color represents upregulated genes, and blue, downregulated genes.

Subsequently, we aimed to identify differentially expressed RNAs between the 6 and 24 h incubation period in each of the experimental conditions separately. In the CTRL group (WPMY1 24–6 h), we observed 124 downregulated and 432 upregulated genes. In the WR1 group (WPMY1 + RWPE1 24–6 h), we observed 327 downregulated and 729 upregulated genes, and in the WR2 group (WPMY1 + RWPE2 24–6 h), 278 and 1021, respectively (Figure 1). Detailed information about DE genes in each condition is presented in Supplementary Table S2.

In what follows, we performed a pathway enrichment analysis based on the unique DE results in the WR1 and WR2 groups, separately. We received 98 and 70 significant pathways for the WR1 and WR2 conditions, respectively (Supplementary Table S3). The top 10 enriched biological processes are presented in Table 1.

### 2.2. Estimation of Transcript Factors Activity

**Figure 2:**
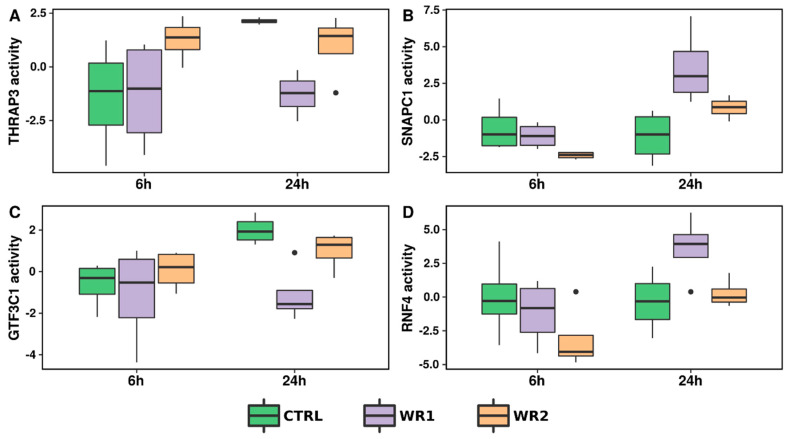
Nominally significant changes of transcription factor (TF) activity in separate comparisons of WR1 vs. WR2 group at 6 (**A**) or 24 h (**B**–**D**) time points. (**A**) THRAP3; (**B**) SNAPC1; (**C**) GTF3C1; (**D**) RNF4.

Based on the mRNA expression profiles from sequencing, we estimated TF activity in each experimental condition. Our analysis did not show any statistically significant TFs with FDR < 0.05 neither at the 6 h, nor the 24 h co-culture. Yet, there were some significant TFs with nominal *p*-value < 0.05. In particular, after 6 h, we found only 4 TFs (1 negatively and 3 positively regulated) in comparison with CTRL and WR2 and 1 negatively regulated TF between WR1 and WR2. After 24 h of co-culture, we found 13 TFs between CTRL and WR1 (7 negatively and 6 positively regulated), 2 positively regulated TFs between CTRL and WR2 and 3 TFs between WR1 and WR2 (1 negatively and 2 positively regulated). Detailed information about the results of these comparisons is in Supplementary Material 4. Since the FDR correction resulted in no significant results, we decided to show only nominally significant results for WR1 vs. WR2 comparisons, as they are most important (Figure 2). Our TF activity analysis suggest that activation of RNF4 and SNAPC1 may be associated with normal tissue growth (co-culture with RWPE1), whereas activation of TFs such as THRAP3 and GTF3C1 could be associated with tumorigenic cells (RWPE2).

**Figure 3:**
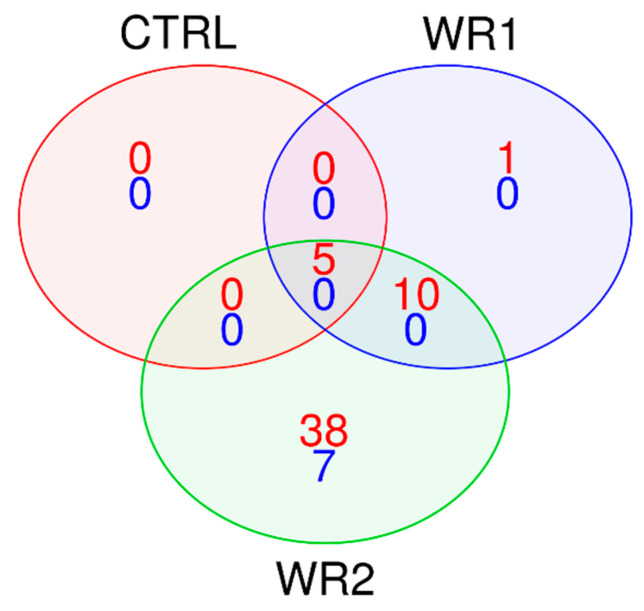
Venn diagram of TFs that changed activity between the 6 and 24 h time points.

For a better estimation of transcription, we took into consideration changes in mRNA expression between the 6 and 24 h time points, to discover significant differences in TF activity. We found 5 TFs for CTRL, 16 TFs for WR1 and 60 TFs for WR2, with significant differences between 6 and 24 h (Supplementary Table S4). Interestingly, those five TFs discovered in CTRL (RUFY3, SCMH1, NFIX, ZBTB25 and NFE2L1) were also presented in co-cultures with RWPE1 or RWPE2 (Figure 3) and were activated at similar levels (Supplementary Table S4). Only one TF, RNF4, was unique for RWPE1; moreover, its activity was increased after 24 h of incubation in WR1 vs. WR2 co-culture. Most of the dysregulated TFs were unique for co-culture with RWPE2 (45 TFs), yet these failed to be differentially activated between the WR1 and WR2 conditions.

The most significant changes were observed after incubation with RWPE2 cells, where 45 TFs were differently activated after 24 h. Since none of these TFs were discovered in 6 or 24 h comparisons, it is more likely that those TFs have different fluctuation/regulation levels of some pathways. Therefore, we performed the Reactome pathway enrichment analysis using these selected TFs. Significant pathways are presented in Table 2.

### 2.3. Validation of lncRNA FER1L4 Expression and miRNA Targets Prediction

**Figure 4:**
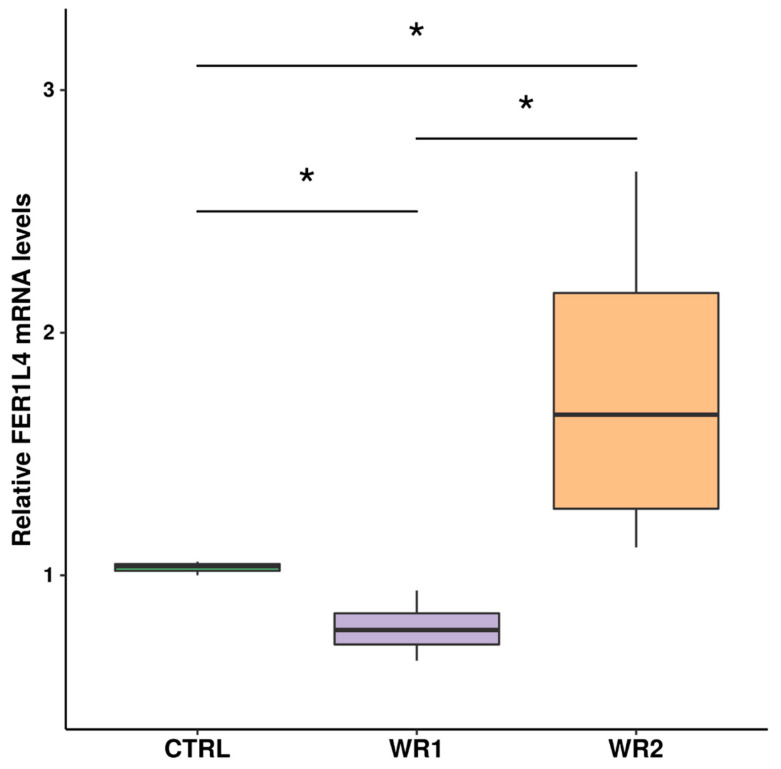
Expression of FER1L4 measured by RT-qPCR in experimental conditions after 24 h of co-culture. (* *p* < 0.05 by Kruskal–Wallis test).

Based on the mRNAseq expression analysis, we discovered overexpression of lncRNA FER1L4 in WPMY1 cell after 24 h co-culture with RWPE2 cells. Therefore, we ought to validate those results with RT-qPCR. After 24 h of co-culture, we observed significant upregulation of FER1L4 expression in the WR2 condition in comparison to the CTRL and WR1 conditions (Figure 4).

Since FER1L4 may, in fact, be responsible for the observed dysregulation in the mRNA expression network by acting as competitive endogenous RNA (ceRNA) and sponging multiple micro RNAs (miRNAs), we decided to identify miRNAs which may interact with *FER1L4*. In the DIANA-LncBase, we found 27 miRNAs with experimentally supported miRNA targets on *FER1L4* transcripts (Supplementary Table S5). Then, we performed a Kyoto Encyclopedia of Genes and Genomes (KEGG) enrichment pathways analysis to identify pathways regulated by those miRNAs (Table 3).

## 3. Discussion

In this study, we investigate changes in TF activity after exposing fibroblasts to normal epithelial cells (RWPE1) or cells with cancer phenotype (RWPE2). The co-culture of fibroblasts with normal epithelial cells (WR1 condition) results in the activation of two TFs, RNF4 and SNAPC1. In the WR2 condition co-culture with the cancerous cell line, activation of GTF3C1 and THRAP3 can be seen. The pathway analysis, performed with Reactome, showed that dysregulated TF activity in CAF transformation could be associated with the PTEN and the RUNX1 associated pathways. Additionally, we explored the potential role of the identified DE gene, *FER1L4*, in sponging multiple miRNAs, that could, at least partially, be responsible for observed transcript expression dysregulation. In general, our observations are consistent with those of other researchers, who showed that interactions of normal fibroblasts with epithelial cells are associated with a proinflammatory response, whereas the co-culture with cancer epithelial cells is associated with transformation and increased motility of fibroblasts.

Two of the discovered TFs, SNAPC1 and GTF3C1, are associated with DNA-binding Pol III regulated transcription [18]. Interestingly, SNAPC1 activity is higher after co-culture with RWPE1 (normal cells), whereas GTF3C1 activity is higher after co-culture with RWPE2 (cancerous cells). Therefore, this could lead to two different transcription patterns associated with DNA-binding Pol III regulated transcription. Possibly, co-culture of fibroblasts with normal epithelial cells and preferential activation by SNAPC1 TF may promote synthesis of small nuclear RNAs [19], which is responsible for the processing of the primary transcription products of split genes, thus allowing precise alignment and correct excision of introns. Conversely, preferential activation of GTF3C1 in co-culture with transformed epithelial cells may lead to increased ribosome biogenesis and protein synthesis [20] with tumor promoting effects. Thus, changes in TF usage during co-culture may represent an effect of trading by fibroblast precision for efficiency in changed co-culture conditions.

Another TF, RNF4, which was activated after co-culture with normal epithelium, is a small ubiquitin-like modifier (SUMO)-targeting RING domain ubiquitin ligase that directly connects the SUMO and the ubiquitin pathways, resulting in the proteasomal degradation of poly-SUMO-modified substrates [21]. RNF4, along with the transcription repressor ZNF278/PATZ, may regulate steroid receptor-dependent transcription activated by androgen receptor [22,23]. Moreover, silencing RNF4 potentiates TGF-β1 expression and activity [24] and TNF-α- and IL-lβ-induced NF-κB activation [25]. At the same time, lung myofibroblast differentiation was shown to be potentiated by TGF-β which can act through TGF-β1/Smad2 and NF-κB signaling pathways [26].

In our study, after incubation with normal epithelium, we observed upregulated expression of MMP-3. The results by Hsieh et al. [27] showed that expression of MMP-3 was lower in CAFs than in fibroblasts derived from normal tissue. Moreover, they suggest that NF-κB may contribute to reactive oxygen species (ROS)-mediated human MMP-3 suppression in CAFs through interaction with ZBP-89 or other transcriptional repressors. In another study, the NF-κB inhibitor, pyrrolidine dithiocarbamate (PDTC), reduced HMGB1-induced TGF-β1 release and fibroblast to myofibroblast differentiation of lung fibroblasts [28], one of the NF-κB targets in THRAP3, a TF which, in our study, was activated after co-culture with RWPE2 cells.

Our pathway analysis of 45 unique TFs differently activated after incubation with RWPE2 cells showed two main pathways that could be associated with CAF transformation. One of the identified pathways is associated with regulation of PTEN transcription and the other with the regulation of RUNX1 associated transcription. Interestingly, one of the DE genes between the WR1 and WR2 conditions was fer-1-like family member 4 pseudogene (lncRNA-FER1L4). It was shown that FER1L4 can act as a sponge for miR-106a-5p and, therefore, regulate the PTEN expression [29]. Further experiments demonstrated that FER1L4 downregulation leads to decreases in both RUNX1 and PTEN mRNA and protein levels, possibly via sponging miR-106a-5p [30]. However, other papers suggest that RUNX1 and PTEN might have opposite effects in TGF-β-induced myofibroblast differentiation. White et al. [31] showed that PTEN inhibits myofibroblast differentiation in vitro, although the effect was abolished after TGF-β exposure. However, the latest studies have shown that the promotion of TGF-β induced myofibroblast differentiation via the downregulation of PTEN may be regulated by miR-216a [32]. On the other side, RUNX1 modulates the cellular response to TGF-β and promotes myofibroblast differentiation in both marrow-derived and tissue-resident mesenchymal stem cells [33]. Lin et al. [34] also showed that RUNX1 can promote fibroblast activation and increase α-SMA and fibronectin expression. Additional analysis showed that FER1L4 can induce dysregulation of pathways discovered in TF analysis by acting as ceRNA. The sponging of miRNAs by FER1L4 was studied in multiple cancers, including prostate [35] and intestine [36], but it was never observed in CAFs. Therefore, this provides a novel insight in the CAF transformation and shows a potential role of FER1L4 in the regulation of the CAF transformation. Several pathways regulated by miRNA sponged by FER1L4, such as the PI3K-Akt signaling pathway and the TGF-beta signaling pathway, relate to the PTEN and RUNX1 pathways from TF analysis. Our study has several limitations—the major one is that there is only one biological replicate in one in vitro model. Moreover, in light of the current results, one may postulate that the two time points chosen for analysis are not sufficient to describe the transcriptional background of the process of transformation from normal epithelial cells to CAFs. Additional functional analyses (e.g., siRNA or use of inhibitors) are necessary to confirm the obtained results.

We cannot exclude the possibility that, due to these limitations, we did not avoid false negative results. However, we hope that extensive and rigorous statistical analyses let us minimize the chance of false positive conclusions.

Our results demonstrated that CAF transformation can be orchestrated by multiple TFs. We showed pathways associated with PTEN and RUNX1 that could be involved in crosstalk between fibroblasts and epithelial cells in healthy tissue, as well as those associated with tumorigenesis. Moreover, we showed that observed initiation of CAF transformation could be orchestrated by lncRNA-FER1L4, which, by sponging multiple miRNAs, may regulate mRNA expression. Although, further investigations are needed to characterize the crosstalk between pathways and their TFs, which might have profound implications to oncogenesis and anticancer therapies. The crosstalk between molecular pathways at the early stage of CAF transformation could lead to the discovery of regulatory points and developing of new methods in the fight against cancer. Moreover, the identification of the signaling pathways that are activated under specific conditions is crucial for precision medicine development.

## 4. Materials and Methods

### 4.1. Cell Lines and Cell Culture

The WPMY1 (human prostatic myofibroblast stromal cell line from the histological normal adult prostate), RWPE1 (human prostatic epithelial cell line derived from the same peripheral zone of the prostate as WPMY1) and RWPE2 (v-Ki-Ras-induced tumorigenic cell line derived from RWPE1) cell lines were obtained from American Type Culture collection—ATCC (CRL-2854, CRL-11609 and CRL-11610, respectively). WPMY1 cells were maintained in Dulbecco’s Modified Eagle’s Medium/Nutrient Mixture F-12 (DMEM/F12, cat.no. 10565018) supplemented with 5% fetal bovine serum (FBS, cat.no. 10500064), and 1% penicillin/streptomycin antibiotic solution (cat.no. 15140122). RWPE1 and RWPE2 cells were maintained in keratinocyte serum-free medium supplemented with 0.05 mg/mL bovine pituitary extract and 5 ng/mL recombinant epidermal growth factor (K-SFM, no. 17005042). Medium was changed every 2–3 days. All materials were purchased from Thermo Fisher Scientific (Waltham, MA, USA) unless mentioned otherwise. Cells were grown in an incubator at a temperature of 37 °C at 5% CO_2_.

### 4.2. Indirect Coculture of WPMY1 and RWPE1 or RWPE2

WPMY1 cells were seeded in 6-well plates at a density of 4 × 105 cells per well in growth medium. RWPE1 and RWPE2 cells were seeded on 0.4-µm pore size tissue-culture inserts formatted for 6-well plates (cat.no. 83.3930.040, Sarstedt, Numbrecht, Germany), at a density of 3 × 10^5^ and 4 × 10^5^ cells per insert, respectively. After 2 days of separate culture, all cells were starved in FBS-free, antibiotic-supplemented DMEM/F12 medium for 24 h. Then, inserts with RWPE1 and RWPE2 cells were added to 6-well plates with WPMY1 cells, for 6 or 24 h. As a control, we used a WPMY1 culture without addition of an insert. As a result, we obtained three experimental conditions: WPMY1 alone, WPMY + RWPE1 (WR1) and WPMY + RWPE2 (WR2), resulting in 6 replicates of each condition per time point.

### 4.3. RNA Isolation and mRNA Sequencing

Total RNA was extracted from cells using the ReliaPrep RNA MiniPrep System (Promega, Madison, WI, USA). RNA quality and integrity were assessed using TapeStation (Agilent, Santa Clara, CA, USA). Four out of 6 replicates with highest RNA integrity values (RIN) and RNA concentrations were used to prepare mRNA sequencing libraries using the Sense mRNA-Seq Library prep Kit v2 (Lexogen, Vienna, Austria) according to the manufacturer’s protocol. Briefly, after RNA normalization, 1.5 µg of RNA was denatured, polyA-selected on magnetic beads and purified. After reverse transcription, cDNA was indexed and amplified in 12 PCR cycles. Twenty-four individual libraries were pooled, at 1.8 pM final concentration, for a 150-cycle NextSeq high-output flow cell. Paired-end sequencing of 2 × 75 bp was performed on a NextSeq 500 platform (Illumina, San Diego, CA, USA).

### 4.4. NGS Data Preparation

Raw Illumina’s Base Call (BCL) files from a paired-end mRNA sequencing run were demultiplexed and converted into fastq files with Illumina Bcl2Fastq v.2.20.0.422 software. The quality of the reads was assessed using FastQC v.11.8. (http://www.bioinformatics.babraham.ac.uk/projects/fastqc/). After removing, with Cutadapt v.1.18 [37], the starter/stopper heterodimer sequence from reads, according to the kit manufacturer’s instructions, we aligned the reads to a reference genome GRCh38, ensembl version 95, with STAR aligner v.2.7.0c [38], using a two-pass method. The reads mapped to each gene were enumerated using HT-Seq-count v.0.11.2 [39] with ensembl version 95 for gene annotation. Quality assessment of alignment and enumerated gene expression levels was performed using MultiQC v.1.9 [40]. Raw expression values provided in a tab-delimited format were then uploaded into R for further analysis. The raw mRNA sequences, along with raw counts from HTSeq software, were deposited in GEO (GSE159493).

### 4.5. mRNA-seq Analysis

The raw reads aligned to particular mRNAs were transformed using the voom function in the package ‘limma’ [41]. Subsequently, two surrogate variables were estimated using the sva package in R. Due to the limited sample size, we set the number of confounders to two. All following differential expression analysis were performed with the ‘limma’ package in R statistical environment on the residuals of the read counts from the linear model with two independent variables (both estimated surrogate variables).

At the beginning, we performed standard pairwise comparisons between WPMY1 cells and co-cultures with RWPE1 or RWPE2 after 6 or 24 h of incubation, separately. Then, to analyze the difference between co-cultures in the context of the time points, we looked at the change in the expression in time in every group to determine which transcripts changed expression in every experimental condition. Therefore, using the lmFit function in ‘limma’, we tested contrasts between time points (24–6 h) in each condition.

### 4.6. TFs Estimation Analysis

TF activity was calculated using the SEPIRA (Systems EPigenomics Inference of Regulatory Activity) package [42] using mRNA expression patterns. First, we constructed a fibroblast-specific network of TFs using GTEx v7 RNA-seq of 483 cultured fibroblasts [43]. Then, we reduced the number of TFs to the top-ranking 100 factors which are involved in regulation of transcription in normal fibroblasts from GTEx. Next, we calculated the TF activity score of the constructed network from mRNA-seq expression data from our samples. Scores with >1 referred to positive regulation, <−1 to negative regulation and from 1 to −1 to no regulation. Differentially activated TFs between time points in every group were estimated with the ‘limma’ package in R statistical environment. To analyze differences between co-cultures in the context of the time points, we used the same contrasts as for mRNA expression.

### 4.7. RT-qPCR Validation of FER1L4 Expression

The lncRNA-FER1L4 expression was validated with RT-qPCR. Reverse transcription was performed with SuperScript III Reverse Transcriptase (Thermo Fisher Scientific, Waltham, MA, USA), according to the manufacturer’s instructions, with 1 ug of RNA and a mix of oligo(dT)20 and random hexamers (2 μM each, E0105-01, E0101-01, EURx, Gdansk, Poland). The generated cDNA template was diluted 10 times. The qPCR reaction was performed in 25 μL volume with Fast Probe qPCR Master Mix master mix (E0422-03, EURx, Gdansk, Poland), 4 μL of cDNA template and 1.25 μL of taqman probes (Hs00957061_m1 for *FER1L4* or Hs02786624_g1 for *GAPDH*, Thermo Fisher Scientific, Waltham, MA, USA). The cycling conditions were 95 °C for 3 min, followed by 40 cycles of 95 °C for 10 s and 60 °C for 30 s. Each qPCR reaction was performed in triplicate. Raw Cq values were processed in CFX Maestro software (Biorad, Hercules, CA, USA). The expression was calculated with the ∆∆Cq method with, *GAPDH* as a reference gene. Statistical analysis was conducted in R.

### 4.8. FER1L4-miRNA Interaction and KEGG Pathway Analysis

The experimentally supported interactions of the FER1L4-miRNA were checked in DIANA-LncBase v3 database [44,45]. Discovered miRNAs where then uploaded to DIANA-mirPath [46] to find targeted genes with the DIANA-microT-CDS algorithm [47] and KEGG pathway enrichment analysis. List of the significant KEGG pathways regulated by miRNAs, which interacted with FER1L4 was compared with previous analysis to find potential common regulatory pathways.

## Figures and Tables

**Table 1:** Top10 gene ontologies (GOs) for gene enrichment analysis of DE genes with logFC > 1 unique for WR1 and WR2 conditions.

Condition	GO.ID	Term	*p*-Value	Annotated_Genes
WR1	GO:0019221	Cytokine-mediated signaling pathway	2.50 × 10^−4^	*CXCL2*, *IL12RB1*, *CD40*, *TRADD*, *CSF3*, *PTPN6*, *GHR*, *TNFRSF8*, *MT2A*, *CD70*, *LCN2*, *MMP3*, *CXCL8*, *PALM3*
	GO:0071222	Cellular response to lipopolysaccharide	8.30 × 10^−4^	*CXCL2*, *CSF3*, *LCN2*, *ANKRD1*, *CXCL8*, *NFKBIL1*
	GO:0015804	neutral amino acid transport	1.22 × 10^−3^	*SLC7A5*, *RGS4*, *SLC6A9*
	GO:0006855	Drug transmembrane transport	2.12 × 10^−3^	*SLC7A5*, *RGS4*, *SLC47A1*, *SLC25A18*
	GO:0071356	Cellular response to tumor necrosis factor	2.51 × 10^−3^	*CD40*, *TRADD*, *TNFRSF8*, *CD70*, *LCN2*, *ANKRD1*, *CXCL8*
	GO:0019724	B cell-mediated immunity	3.50 × 10^−3^	*CD40*, *PTPN6*, *CD70*, *C4A*
	GO:0009605	Response to external stimulus	3.59 × 10^−3^	*TYMP*, *TNC*, *ASNS*, *CXCL2*, *IGSF9*, *IL12RB1*, *PCK2*, *CD40*, *TRADD*, *CORO1A*, *AKNA*, *CSF3*, *STC2*, *NR4A3*, *TNFRSF8*, *MT2A*, *NPAS1*, *MYPN*, *LYG1*, *EPHA1*, *LCN2*, *ANKRD1*, *MMP3*, *MARS*, *CXCL8*, *NUPR1*, *PALM3*, *NFKBIL1*, *C4A*
	GO:0019730	Antimicrobial humoral response	3.67 × 10^−3^	*CXCL2*, *LCN2*, *CXCL8*
	GO:0061469	Regulation of type B pancreatic cell proliferation	4.12 × 10^−3^	*NR4A3*, *NUPR1*
	GO:0032354	Response to follicle-stimulating hormone	4.12 × 10^−3^	*ASNS*, *PAPPA*
WR2	GO:0001541	Ovarian follicle development	3.30 × 10^−5^	*DMC1*, *LFNG*, *VGF*, *SOD1*, *KIT*
	GO:0045987	Positive regulation of smooth muscle contraction	6.50 × 10^−4^	*TACR2*, *GHRL*, *KIT*
	GO:0060285	Cilium-dependent cell motility	9.90 × 10^−4^	*DNAH7*, *CFAP54*, *CFAP57*
	GO:0003341	Cilium movement	3.67 × 10^−3^	*DNAH7*, *MAATS1*, *CFAP54*
	GO:0042745	Circadian sleep/wake cycle	3.89 × 10^−3^	*GHRL*, *NLGN1*
	GO:0099175	Regulation of post-synapse organization	4.04 × 10^−3^	*GHRL*, *NLGN1*, *GAP43*, *FZD9*
	GO:0045747	Positive regulation of Notch signaling pathway	4.29 × 10^−3^	*LFNG*, *KIT*, *MESP1*
	GO:1900029	Positive regulation of ruffle assembly	4.64 × 10^−3^	*CARMIL2*, *NLGN1*
	GO:0045986	Negative regulation of smooth muscle contraction	4.64 × 10^−3^	*CALCRL*, *SOD1*
	GO:0051290	Protein heterotetramerization	4.96 × 10^−3^	*NLGN1*, *S100A10*, *HIST1H4A*

GO.ID—The Gene Ontology Identifier.

**Table 2:** Reactome analysis of unique TFs for 6 vs. 24 h time points in the WR2 condition.

Pathway Identifier	Pathway Name	Entities *p*-Value	Entities FDR	TFs
R-HSA-8953750	Transcriptional Regulation by E2F6	2.14 × 10^−5^	2.66 × 10^−3^	YAF2, EED, CBX5
R-HSA-8939243	RUNX1 interacts with co-factors whose precise effect on RUNX1 targets is not known	3.55 × 10^−4^	2.20 × 10^−2^	YAF2, SMARCE1, SMARCA2
R-HSA-8943724	Regulation of PTEN gene transcription	1.90 × 10^−3^	4.68 × 10^−2^	EED, REST, SNAI2
R-HSA-3247509	Chromatin modifying enzymes	1.95 × 10^−3^	4.68 × 10^−2^	SMARCE1, EED, SMARCA2, REST, KDM5A
R-HSA-4839726	Chromatin organization	1.95 × 10^−3^	4.68 × 10^−2^	SMARCE1, EED, SMARCA2, REST, KDM5A

FDR—false discovery rate; TFs—transcription factors.

**Table 3:** The top 10 KEGG pathways enriched by the target genes of 27 micro RNAs (miRNAs) with experimentally supported interaction with FER1L4.

KEGG Pathway	*p*-Value	FDR	Number of Genes	Number of miRNAs
ECM-receptor interaction (hsa04512)	6.09 × 10^−20^	1.23 × 10^−17^	11	18
Signaling pathways regulating pluripotency of stem cells (hsa04550)	6.91 × 10^−6^	6.97 × 10^−4^	18	22
Mucin type O-Glycan biosynthesis (hsa00512)	6.62 × 10^−5^	3.63 × 10^−3^	4	12
Focal adhesion (hsa04510)	8.23 × 10^−5^	3.63 × 10^−3^	23	20
PI3K-Akt signaling pathway (hsa04151)	9.13 × 10^−5^	3.63 × 10^−3^	32	23
Lysine degradation (hsa00310)	1.08 × 10^−4^	3.63 × 10^−3^	5	24
Glioma (hsa05214)	2.14 × 10^−4^	6.16 × 10^−3^	8	19
Glycosphingolipid biosynthesis—lacto and neolacto series (hsa00601)	2.95 × 10^−4^	7.46 × 10^−3^	3	12
Adipocytokine signaling pathway (hsa04920)	6.49 × 10^−4^	1.24 × 10^−2^	10	17
TGF-beta signaling pathway (hsa04350)	7.09 × 10+	1.24 × 10^−2^	10	19
Protein digestion and absorption (hsa04974)	7.10 × 10^−4^	1.24 × 10^−2^	12	19
MAPK signaling pathway (hsa04010)	7.37 × 10^−4^	1.24 × 10^−2^	23	23
Nicotine addiction (hsa05033)	1.05 × 10^−3^	1.63 × 10^−2^	5	17
AMPK signaling pathway (hsa04152)	1.55 × 10^−3^	2.24 × 10^−2^	13	17
mTOR signaling pathway (hsa04150)	1.75 × 10^−3^	2.36 × 10^−2^	9	18
ErbB signaling pathway (hsa04012)	1.96 × 10^−3^	2.48 × 10^−2^	10	21
Amoebiasis (hsa05146)	2.23 × 10^−3^	2.63 × 10^−2^	7	13
Melanoma (hsa05218)	2.35 × 10^−3^	2.63 × 10^−2^	9	19
Proteoglycans in cancer (hsa05205)	2.69 × 10^−3^	2.86 × 10^−2^	16	20
Pancreatic cancer (hsa05212)	4.09 × 10^−3^	4.03 × 10^−2^	8	18
Wnt signaling pathway (hsa04310)	4.19 × 10^−3^	4.03 × 10^−2^	12	21
Cytokine-cytokine receptor interaction (hsa04060)	4.52 × 10^−3^	4.15 × 10^−2^	14	20

FDR—false discovery rate.

## Supplementary Materials

Supplementary Materials can be found at https://www.mdpi.com/1422-0067/21/22/8749/s1. Supplementary Table S1. DE genes between WPMY1 cells and WPMY1 cocultured with RWPE1; Supplementary Table S2. DE genes between 6 h and 24 h incubation time points in each of the experimental conditions; Supplementary Table S3. Significant topGO pathways (BP) for unique DE genes in WR1 and WR2 conditions separately; Supplementary Table S4. Statistically significant TFs activity between 6 h and 24 h incubation time points in each of the experimental conditions. Supplementary Table S5. Experimentally validated interactions of FER1L4 with miRNAs in DIANA-LncBase v3.

## Abbreviations

α-SMA	Alpha smooth muscle actin
ceRNA	Competitive endogenous RNA
CAFs	Cancer-associated fibroblasts
CXCL12	Stromal cell-derived factor 1(SDF1)
ECM	Extracellular matrix
EGFR	Epidermal growth factor receptor
ERK	Extracellular signal regulated kinase
FER1L4	Fer-1 like family member 4
IL-1	Interleukin 1
lncRNA	Long non-coding RNA
TNF-α	Tumor necrosis factor alpha
RUNX1	Runt related transcription factor 1
TGF-β	Transforming growth factor beta
